# Predicting pediatric Crohn's disease based on six mRNA-constructed risk signature using comprehensive bioinformatic approaches

**DOI:** 10.1515/biol-2022-0731

**Published:** 2023-10-05

**Authors:** Yuanyuan Zhan, Quan Jin, Tagwa Yousif Elsayed Yousif, Mukesh Soni, Yuping Ren, Shengxuan Liu

**Affiliations:** Department of Plastic and Cosmetic Surgery, Tongji Hospital, Tongji Medical College, Huazhong University of Science and Technology, 1095 Jiefang Avenue, Wuhan 430030, China; Department of Rehabilitation, Xiantao First People’s Hospital Affiliated to Yangtze University, Xiantao 433099, Hubei, China; Department of Medical Laboratory Technology, Faculty of Applied Medical Sciences, Jazan University, Gizan, 45142, Saudi Arabia; Department of CSE, University Centre for Research & Development, Chandigarh University, Mohali, Punjab – 140413, India; Department of Pediatrics, Tongji Hospital, Tongji Medical College, Huazhong University of Science and Technology, 1095 Jiefang Avenue, Wuhan 430030, Hubei, China

**Keywords:** pediatric Crohn’s disease, risk signature, diagnosis, prediction, immune

## Abstract

Crohn’s disease (CD) is a recurrent, chronic inflammatory condition of the gastrointestinal tract which is a clinical subtype of inflammatory bowel disease for which timely and non-invasive diagnosis in children remains a challenge. A novel predictive risk signature for pediatric CD diagnosis was constructed from bioinformatics analysis of six mRNAs, adenomatosis polyposis downregulated 1 (APCDD1), complement component 1r, mitogen-activated protein kinase kinase kinase kinase 5 (MAP3K5), lysophosphatidylcholine acyltransferase 1, sphingomyelin synthase 1 and transmembrane protein 184B, and validated using samples. Statistical evaluation was performed by support vector machine learning, weighted gene co-expression network analysis, differentially expressed genes and pathological assessment. Hematoxylin–eosin staining and immunohistochemistry results showed that APCDD1 was highly expressed in pediatric CD tissues. Evaluation by decision curve analysis and area under the curve indicated good predictive efficacy. Gene Ontology, Kyoto Encyclopedia of Genes and Genomes and gene set enrichment analysis confirmed the involvement of immune and cytokine signaling pathways. A predictive risk signature for pediatric CD is presented which represents a non-invasive supplementary tool for pediatric CD diagnosis.

## Graphical abstract

**Figure:**
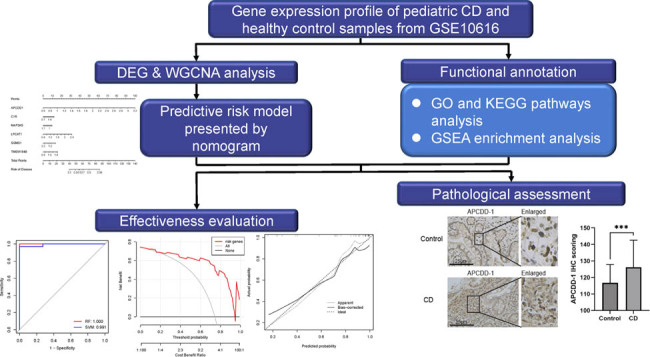

## 1 Introduction

Crohn’s disease (CD) is a highly heterogeneous, recurrent chronic inflammatory condition of the gastrointestinal tract and is a clinical subtype of inflammatory bowel disease (IBD). The incidence of pediatric CD has shown a steep increase over the last 30 years [1], particularly in China since 2000 [2,3], and one in four patients diagnosed with IBD was under the age of 18. Clinical manifestations of pediatric CD tend to be more severe and active than those of adult CD, and long-term consequences may include growth retardation [4,5]. Early diagnosis improves treatment outcomes, as cure rates and treatment tolerance are better in younger children [6]. Thus, there is a need for additional diagnostic tools in the form of novel biomarkers to assist with timely and accurate diagnosis. Diagnoses depend on the assessment of clinical manifestations, laboratory tests, endoscopy, imaging and histopathology, but early clinical manifestations of nausea, vomiting, fever, abdominal pain and distention are nonspecific and atypical, making distinction from other abdominal diseases difficult [7]. Pediatric CD also produces nonspecific laboratory, endoscopic, radiological and histological findings. Therefore, diagnostic tools using novel biomarkers are essential for the early detection of pediatric CD.

Predictive risk signatures have been increasingly utilized for disease diagnosis, and pediatric CD pathogenesis is known to be related to individual gene profile variation [8] and expression of risk genes, especially in younger children [9,10]. Geographical variation is also seen in genetic predisposition to pediatric CD, and common mutations of nucleotide oligomerization domain (*NOD*2)/CARD15 present in Caucasian patients have not been identified in Asian Han and Zhuang populations [11,12]. Susceptibility loci studies, including multi-ethnic, multi-center cohort and whole-genome sequencing analysis, have identified predictive variants for IBD [13,14]. Ethnicity appears to influence susceptibility site outcomes, and mutations of lipopolysaccharide-responsive beige-like anchor protein and cytotoxic T lymphocyte-associated protein 4 have been indicated as rare variants related to early-onset IBD, especially in African American populations [14]. Some individual genetic markers of CD and IBD have been suggested for their diagnostic, prognostic and therapeutic value, but single genes give little information about the disease, and models composed of multiple genes may constitute a superior reflection of clinical features. However, little has been reported regarding predictive models for pediatric or adult CD.

Machine learning (ML), including random forest (RF), support vector machine (SVM), K-nearest neighborhood, and Naïve Bayes approaches, have been employed in social sciences and medical research. ML algorithms are flexible and scalable, making them suitable for diagnosis and risk stratification [15], with varying predictive power depending on the task and data type. A recent systematic review indicated the superiority of RF for disease prediction [16], and the non-invasive nature of ML makes it a promising auxiliary diagnostic tool for IBD.

A predictive risk signature was constructed from novel mRNA markers identified by weighted gene co-expression network analysis (WGCNA) and differentially expressed genes (DEGs) for pediatric CD diagnosis and validated by pathological assessment. The robustness of the current predictive risk signature and potential as a diagnostic tool for pediatric CD are demonstrated.

## 2 Materials and methods

### 2.1 Biopsy samples

Pediatric CD patients were recruited through the Pediatric Gastroenterology service of Tongji Hospital during 2021–2022. Written informed consent was given by all participants, and ethical approval was granted by the Tongji Hospital Ethics Board (TJ-IRB20220756). All patients were under 17 and assigned to a CD group, diagnosed according to the modified Porto criteria [17] with diagnosis confirmed by endoscopic biopsy or a control group of patients who were not diagnosed with CD. Tissue biopsies were collected by gastrointestinal endoscopy from gastrointestinal tract lesion sites and stained for pathological assessment. Detailed patient information is given in Table S1.


**Informed consent:** Informed consent was obtained from all individuals included in this study.
**Ethical approval:** The research related to human use has been complied with all the relevant national regulations and institutional policies and in accordance with the tenets of the Helsinki Declaration, and has been approved by the Tongji Hospital Ethics Board (TJ-IRB20220756).

### 2.2 Bioinformatics data information

The gene expression profile of dataset GSE10616, including 10 ulcerative colitis (UC), 13 colon-only CD, 18 ileocolonic CD and 11 normal samples, from the GEO online database (https://www.ncbi.nlm.nih.gov/geo/) and annotated by the platform Affymetrix GeneChip Human Genome U133 Plus 2.0 Array. Clinical information related to the dataset is given in Table S2.

### 2.3 Data preprocessing and DEG analysis

A total of 9,989 probes in the GSE10616 dataset were normalized and DEGs between CD and normal samples were identified using “limma” R package in R.

### 2.4 Establishment of weighted co-expressed gene modules

WGCNA is a well-established bioinformatics approach for building scale-free networks and conducting module analysis using gene expression profiles and was performed using the “WGCNA” and “pheatmap” R packages in R. Unavailable data and clinical subtypes of the dataset GSE10616 were eliminated to ensure that gene expression profiles from pediatric CD and healthy control samples only were included. A scale-free network was formed, and the most suitable soft threshold (*β* = 13) was determined according to the computed average connection. A topological overlap matrix (TOM) and corresponding dissimilarity TOM (dissTOM) were computed, and hierarchical clustering, based on dissTOM, was carried out in the form of a systematic clustering dendrogram. mRNAs from GSE10616 were categorized into 12 modules of 12 different colors with a minimum module size of 100. The correlation between clinical traits and module eigengenes in each module was calculated.

### 2.5 ML model development and construction of a nomogram

Two-stage selection was performed to identify the most relevant mRNAs for inclusion. Candidate mRNAs were selected from the intersection of DEGs and WGCNA hub genes and used to construct RF and SVM learning signatures. Five-fold cross-validation was used to verify the predictive performance of the signatures in the training cohort, and performance was evaluated by cumulative residual distribution analysis, boxplot distribution analysis and receiver operating characteristic (ROC) curve.

A nomogram shows the occurrence probability of individual clinical diagnosis through aggregating scores of multiple predictor variables and is a frequently used tool used in the calculation of disease diagnosis risk. A nomogram to predict the risk of pediatric CD diagnosis was constructed based on the DEGs and WGCNA analysis by the “rms” R package. Calibration plots and area under the curve were also calculated to assess nomogram accuracy in predicting risk.

### 2.6 Pathway enrichment analysis

The mRNAs in GSE10616 were analyzed by gene set enrichment analysis (GSEA) 4.2.1 software Kyoto Encyclopedia of Genes and Genomes (KEGG) pathway analysis performed and visualized by “clusterProfiler,” the “pathview” and the “ggplot2” R package to identify pathways enriched in DEGs between control and CD samples. DEGs were assigned to pathways by gene ontology (GO) analysis, visualized by “clusterProfiler,” the “topGO” and the “ggplot2” R package.

### 2.7 Hematoxylin–eosin staining (H&E) and immunohistochemistry (IHC) assay

Biopsy tissues were fixed with 4% paraformaldehyde for 48 h and covered with paraffin for sectioning into 4 μm sections and H&E staining (Servicebio, G1003, China) for histological analysis.

IHC of colon sections from lesion sites was performed to investigate the expression of adenomatosis polyposis downregulated 1 (APCDD1). Colon tissues were immersed in 4% paraformaldehyde for 24 h, embedded in paraffin, and sectioned and antigen retrieval was performed by boiling slides for 20 min in ethylene diamine tetraacetic acid citrate buffer (pH = 6.0). Sections were incubated with anti-APCDD1 antibody (Signalway antibody, 43286, China, 1:50) at 4°C overnight, washed with PBS/Tween20 buffer (pH = 7.4) and anti-rabbit secondary antibody added for 50 min at room temperature. The 3,3′-diaminobenzidine chromogenic reagent kit was used to visualize staining under the microscope (SOPTOP, CX40, China), and samples were analyzed by ImageJ software (NIH, bundled with 64-bit Java 1.8.0_172).

### 2.8 Statistical analysis

All data are expressed as mean  ±  SEM with Student’s *t*-test performed to determine statistical differences between groups. A value of *p*  <  0.05 was considered statistically significant.

## 3 Results

### 3.1 Identification of differentially expressed mRNAs in pediatric CD and normal colon tissues

**Figure 1:**
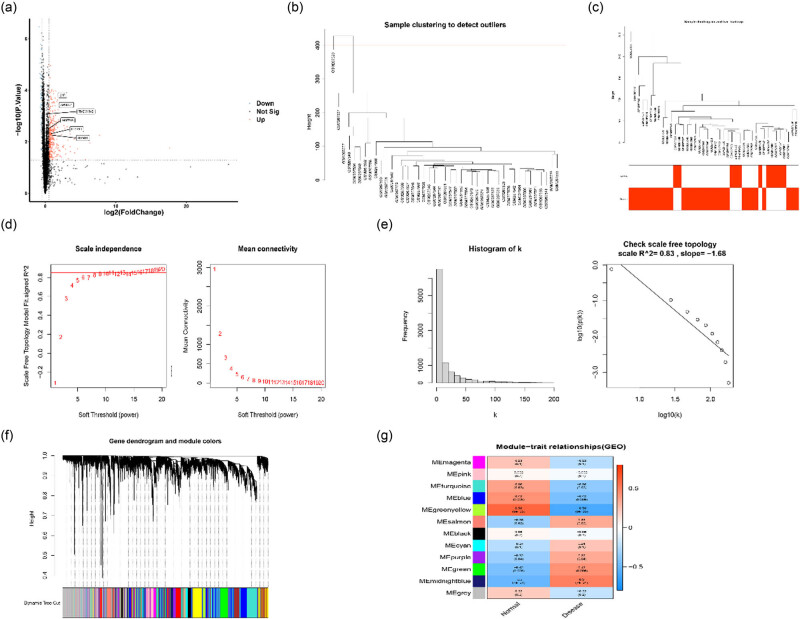
DEGs and WGCNA analysis of pediatric CD and control groups (a) Volcano plot for mRNAs based on *p*-value < 0.05 and |log FC| > 0.5. (b) An outlier was detected when the critical value was 400. (c) Sample tree and corresponding clinical traits. Clustering was visualized by Pearson correlation coefficient calculation. (d) Identification of the best soft threshold for WGCNA. The mean connectivity was the best with a soft threshold of 13. (e) Construction of connection distribution histogram and checking of range-free topology (*β* = 13). (f) Dendrogram of all genes clustered utilizing a dissimilarity measure (1-TOM). (g) Identification of 12 modules related to the clinical traits of pediatric CD.

A total of 561 DEGs with *p* <  0.05 and |log Fold Change| (|log FC|)  >  0.5 were identified from GSE10616, including 376 upregulated and 185 downregulated, and are shown in the volcano plot (Figure 1a).

### 3.2 Weighted co-expression network construction and clinically significant module analysis

The weighted co-expression network was constructed utilizing the gene expression matrix and clinical information in dataset GSE10616 through the R package of “WGCNA.” Data scrubbing of the expression profile was performed to exclude unavailable data and clinical subtypes, including “Internal control” and “Ulcerative colitis,” to ensure the inclusion of only suitable samples and genes. One outlier sample (GSM267529) was screened out by Pearson correlation analysis with a height threshold of 400 (Figure 1b) to ensure the stability of the co-expression network. Sample clustering with clinical traits was established (Figure 1c), and the power of *β* = 13 was selected to be the soft-thresholding parameter to guarantee a scale-free network (Figure 1d and e). A hierarchical clustering tree was constructed to complete the recognition of the co-expression network (Figure 1f). Twelve gene modules were identified with a minimum module size of 100, and the correlation between clinical traits and modules was assessed by analyzing the eigengene of each module. The midnight blue module had the strongest correlation (*r* = 0.5, *p* = 7 × 10^−4^) with clinical traits (Figure 1g) and was selected for bioinformatics analysis of genes and DEGs.

A total of 283 mRNAs with the highest connectivity (gene significance >0.2 and module eigengene-based connectivity [datKME] >0.8) were defined as hub mRNAs, considered to have the greatest degree of association with pediatric CD onset, and were recruited into the hub mRNAs of the midnight blue module (Table S3).

### 3.3 Construction of predictive risk signature

**Figure 2:**
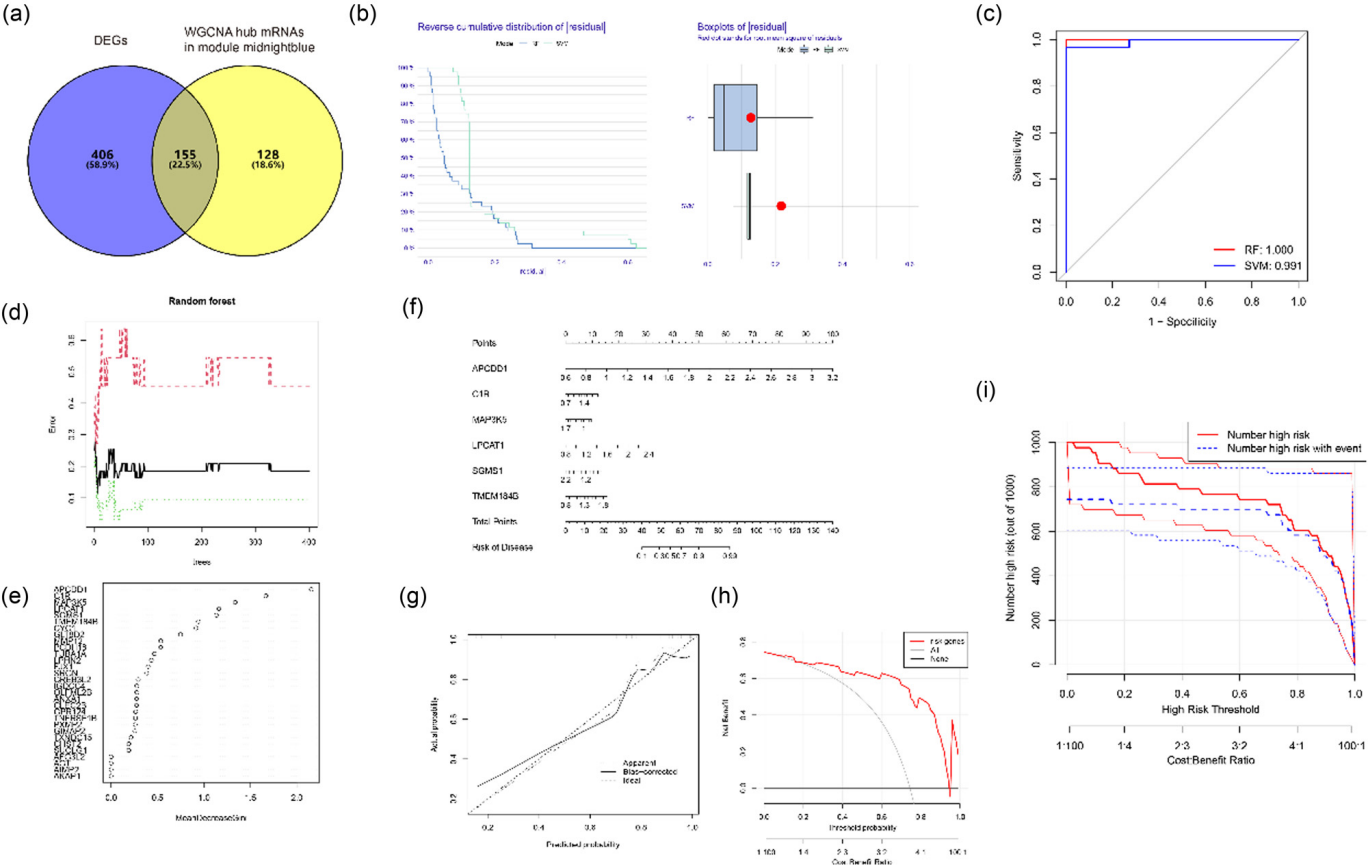
Establishment of a nomogram for risk prediction using the intersection between DEGs and the most clinically associated module in the WGCNA. (a) Intersection of hub mRNAs with DEGs, containing 155 mRNA symbols. (b) Cumulative residual distribution analysis and boxplot distribution analysis of RF and SVM. (c) ROC curve of RF and SVM. (d) Construction of RF model. (e) Gene significance of the top 30 candidate mRNAs. (f) Nomogram combining signatures with clinical traits. (g) Calibration curves comparing nomogram-predicted survival probabilities and measured survival probabilities of training and validation cohorts. *y*-axis: measured survival probabilities; *x*-axis: nomogram-predicted survival probabilities; dotted line: ideal prediction by an optimal signature; solid line: nomogram performance. (h) DCA of predicted nomogram signature. (i) Clinical impact curve.

The intersection of midnight blue hub mRNAs and DEGs, containing 155 gene symbols (Figure 2a). The intersection of the genes with *p* < 0.05 and |logFC| > 0.1 and the midnight blue hub mRNAs contained 199 mRNAs. By using these 199 mRNAs, a quantitative predictive signature assessment of pediatric CD risk. Six independent predictors were included *APCDD*1, *complement component* 1*r* (*C*1*R*)*, mitogen-activated protein kinase kinase kinase* 5 (*MAP*3*K*5)*, lysophosphatidylcholine acyltransferase* 1 (*LPCAT*1)*, sphingomyelin synthase 1* (*SGMS*1) and *transmembrane protein* 184*B* (*TMEM*184*B)*. RF was shown to have the best performance through cumulative residual distribution analysis, boxplot distribution analysis (Figure 2b) and ROC curve (Figure 2c). Risk signature discrimination was calculated using a bootstrap-corrected C statistic, and the area under the ROC curve was 0.991. The top 30 candidate mRNAs were identified from the RF model and gene significance plot (Figure 2d and e) and a nomogram for quantitative predictive signature assessment of pediatric CD risk constructed from the six independent predictors by RF (Figure 2f). The *p* values, logFC and average gene expression rates are shown in Table S4, and risk estimates are generated by aggregation of scores of each predictor in the nomogram. The closeness of the solid and dotted lines indicates the accuracy of the prediction, and the predicted risk was shown to match the incidence by the calibration curve (Figure 2g). A decision curve analysis (DCA) diagram was plotted to refine the predictive signature, and the predictive signature showed good net benefit across the threshold probability range: 0.2–0.9 (Figure 2h). The clinical impact curve was plotted using the signature (Figure 2i), and good potential facilitation of clinical practice shown.

*C*1*R, MAP*3*K*5*, LPCAT*1 and *SGMS*1 have previously been linked to IBD, but any association of *APCDD1* and *TMEM184B* with IBD has not been reported [18,19,20,21]. Differential expression of the six predictor mRNAs between pediatric CD and healthy intestinal tissue from dataset GSE10616 was visualized in the volcano plot (Figure 1a).

### 3.4 Pathway enrichment analysis

**Figure 3:**
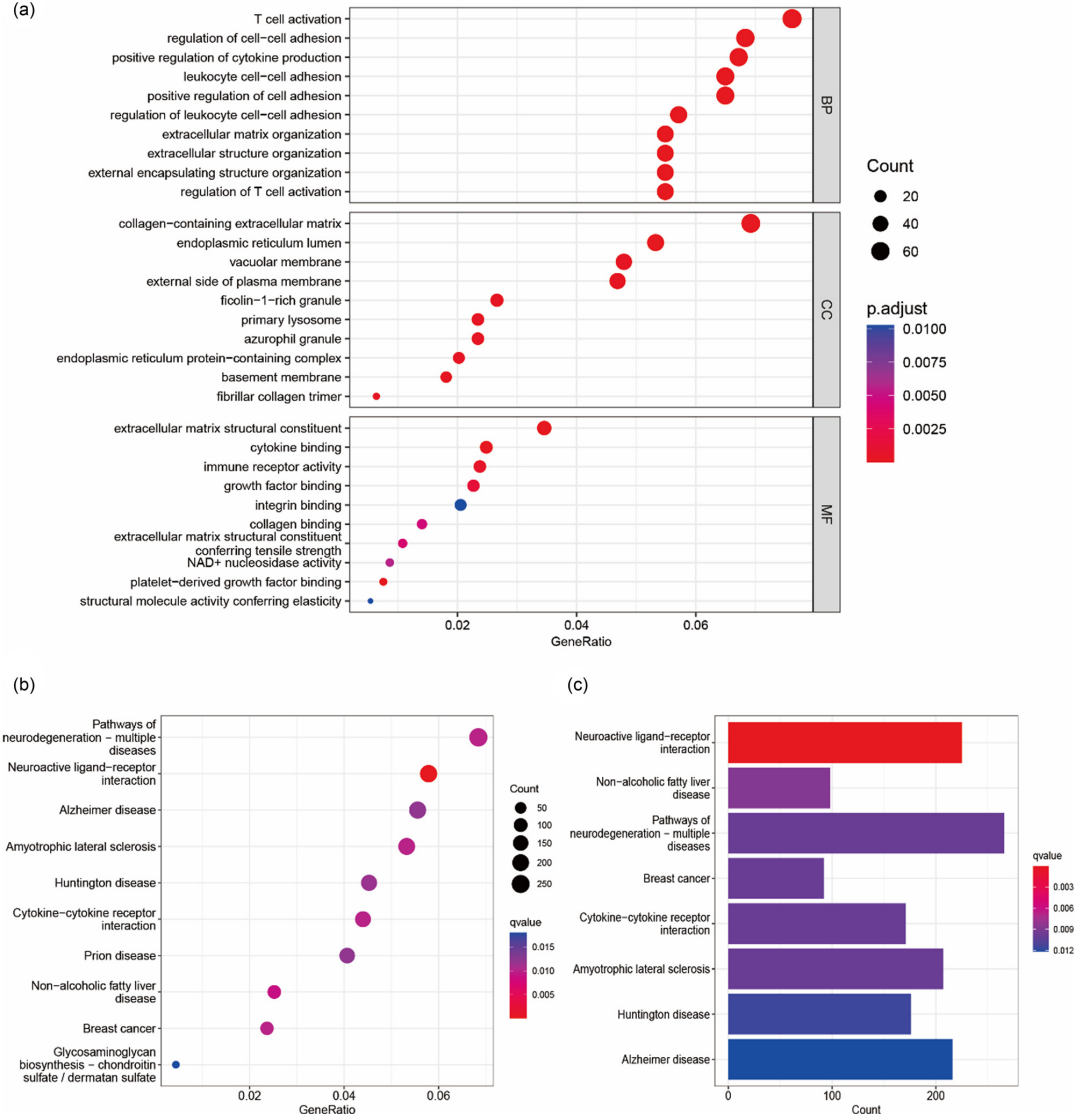
GO and KEGG pathway enrichment analysis of the intersected genes in pediatric CD and control groups. (a) GO analysis of the intersected genes by BP, CC and MF. *y*-axis: GO terms; *x*-axis: gene ratio of each GO term. (b) KEGG pathway enrichment analysis of the intersected genes. *y*-axis: KEGG terms; *x*-axis: gene ratio of each KEGG term. (c) KEGG pathway enrichment analysis of the intersected genes. *y*-axis: KEGG terms; *x*-axis: gene counts of each KEGG term.

Pathway enrichment analysis was performed on the 199 intersecting mRNAs to associate affected pathways with pediatric CD. GO enrichment analysis indicated biological processes (BP) to include T-cell activation, leukocyte cell–cell adhesion, positive regulation of cytokine production and extracellular matrix organization; cellular components (CC), signaling pathways, such as collagen-containing extracellular matrix and azurophil granule (Figure 3a) and molecular functions (MF), cytokine binding, immune receptor activity, growth factor activity, integrin binding and extracellular matrix constituent (Figure 3a). These findings are consistent with the immune and autoimmune implications of IBD. Immune cell infiltration analysis of dataset GSE10616 revealed increased infiltration of plasma cells and M1 macrophages (Figure S1). Increased collagen secretion and remodeling are pathophysiological components of the intestinal strictures and fistulas associated with CD, especially at the chronic stage [22]. KEGG analysis of the pediatric CD group indicated enrichment of cytokines and associated pathways (Figure 3b and c).

**Figure 4:**
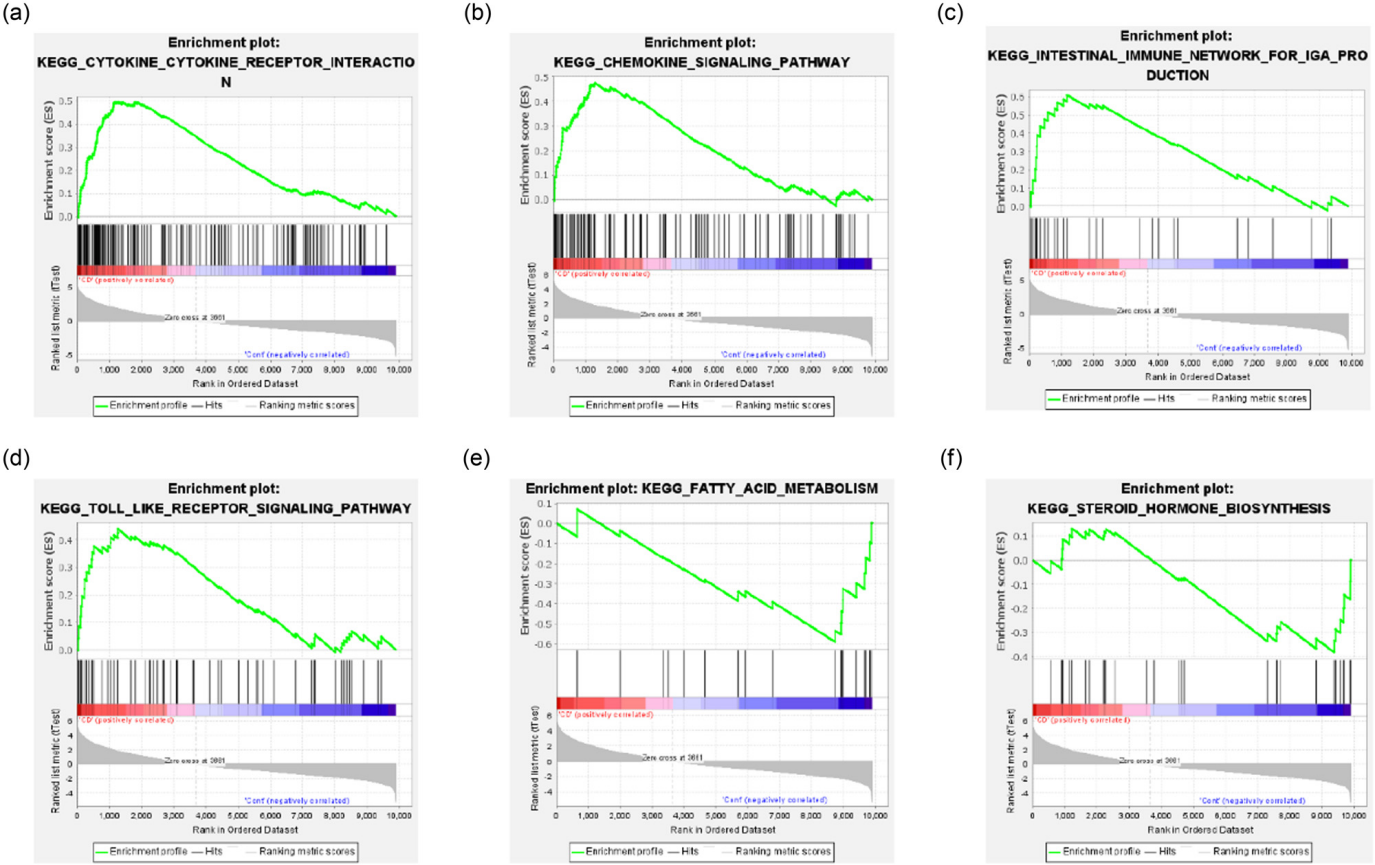
GSEA analysis of genes in pediatric CD and healthy controls from the GSE10616 dataset. CD group genes were enriched in pathways including (a) cytokine-cytokine receptor interaction, (b) chemokine signaling pathways, (c) intestinal immune network for IgA production and (d) toll-like receptor signaling pathway. Healthy control group genes were enriched in pathways related to (e) fatty acid metabolism and (f) steroid hormone biosynthesis.

GSEA showed pediatric CD samples to be enriched for pathways, such as cytokine–cytokine receptor interaction, chemokine signaling pathways, the intestinal immune network for IgA production and toll-like receptor signaling pathway (Figure 4a–d), indicating the possibility that mycobiota orchestrate gut fungal commensalism by inducing IgA antibodies [23]. Control samples were enriched for pathways related to fatty acid metabolism and steroid hormone biosynthesis (Figure 4e and f). Small intestinal epithelial cells from CD patients have previously shown evidence of impaired lipid peroxidation metabolism, and the abundant polyunsaturated fatty acid content of the Westernized diet may lead to enteritis [24]. The top 20 enriched pathways and genes are shown in Table S5.

### 3.5 H&E staining, endoscopic evaluation and IHC validation of APCDD1 overexpression in pediatric CD lesion sites

**Figure 5:**
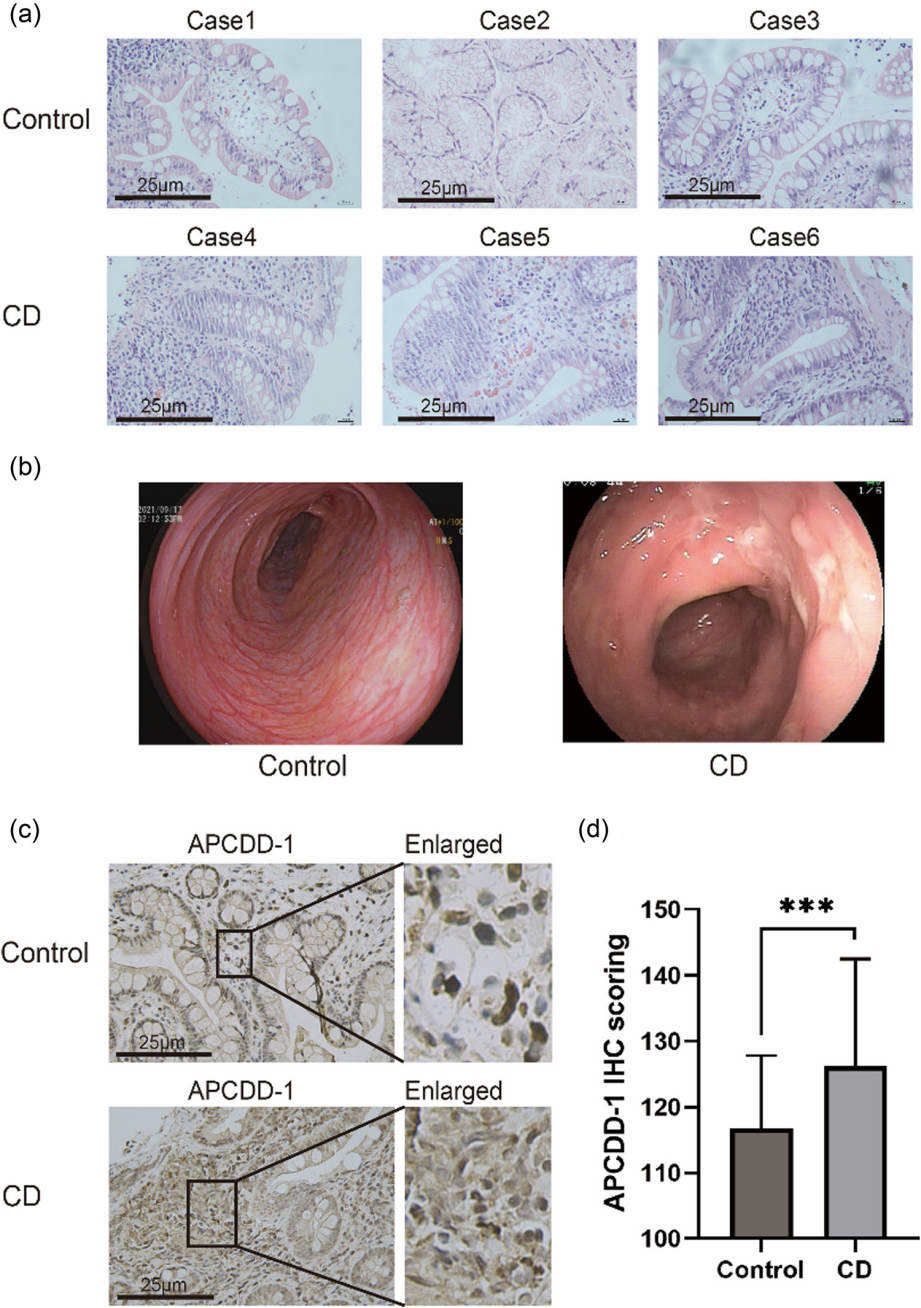
H&E staining, endoscopic views and IHC validation of APCDD1 overexpression in pediatric CD lesion sites (a) H&E staining of control and CD samples (magnification ×400, scale bar = 25 μm). (b) Endoscopic views of control and CD groups. (c) Expression of APCDD1 in control and CD samples (*n* = 5, magnification ×400, scale bar = 25 μm). (d) Quantification of APCDD1 expression in healthy control and CD samples. Case1: terminal ileum; Case2: duodenal bulb; Case3: terminal ileum; Case4: descending colon; Case5: terminal ileum; Case6: descending colon. ***, *p* < 0.001.

H&E staining of CD gastrointestinal biopsies showed damage, including shortening of the intestinal villi, inflammatory cell infiltration and lymphoid follicular formation, compared with controls (Figure 5a). Multiple ulcer lesion sites could be seen during endoscopic examination of pediatric CD patients compared with normal mucosa in controls (Figure 5b).

IHC staining showed significant overexpression of *APCDD*1 in CD lesion tissues compared with controls, consistent with the results of bioinformatics analyses (Figure 5c and d).

## 4 Discussion

The pediatric CD is an increasing global health problem with rising rates in China over the last 20 years due to risk factors, such as the adoption of a Westernized diet and industrial environments. Early diagnosis of pediatric CD is likely to shorten the course of therapy and improve prognosis, but existing diagnostic methods rely on judgment of symptoms, laboratory findings and imaging features. Patients have often entered the chronic and recurring inflammation stage prior to diagnosis to the detriment of treatment [25], illustrating the need for complementary diagnostic tools and early screening tools.

Causes of pediatric CD are multifactorial, including gene mutation, immunity and environmental factors, with the first two being considered more significant [26]. Indeed, gene mutations and positive family history often lead to diagnosis at a younger age and a higher incidence of extra-intestinal manifestations.

Previous studies of predictive risk signatures for pediatric IBD have focused on complications and drug reactions, and some have shown correlations with pathways or molecules. There are a few studies on risk loci for pediatric IBD, but none has previously established a predictive risk signature for the diagnosis of pediatric CD.

The current study identified biomarkers from the gene expression profiles of dataset GSE10616 in the GEO database to establish a predictive risk signature. The midnight blue module from WGCNA analysis had the lowest *p*-value, and intersection with DEGs allowed a nomogram based on six mRNAs, four of which have been previously linked to IBD, to be constructed. *APCDD*1 had the highest gene significance in the risk signature and is an inhibitor of the Wnt/β-catenin signaling pathway and the target of the β-catenin/T-cell factor 4 complex with probable involvement in colorectal carcinogenesis [10]. The locus is a known variant in familial colorectal cancer patients [27] but has not previously been associated with IBD. *C*1*R* encodes a proteolytic subunit in the complement system C1 complex of the innate immune response and is involved in CD progression. The C1R is bound by glucocorticoid receptors and is a potential serum biomarker of response to glucocorticoid treatment in pediatric IBD [18]. *LPCAT*1 encodes an enzyme that converts lysophosphatidylcholine to phosphatidylcholine to regulate the number and size of lipid droplets and has been suggested as an indicator of UC remission [28]. *SGMS*1 was a predicted target of baicalin in a study of the baicalin sphingolipid-linked treatment of colitis [29]. *TMEM*184*B* encodes transmembrane protein 184B, a putative mitogen-activated protein kinase (MAPK)-activating protein, and was upregulated in antineutrophil cytoplasmic antibody-associated glomerulonephritis [30]. MAP3K5 or ASK1 was shown to induce c-Jun N-terminal kinase 1 (JNK/MAPK8/SAPK1) phosphorylation, leading to Bcl-2 activation of cell autophagy, and was associated with endoplasmic reticulum stress, making it a promising therapeutic target for IBD [20]. *MAP*3*K*5 is associated with autophagy-induced ER stress in IBD patients, allowing bacterial colonization of intestinal mucosa [31]. The current study showed that these six predictor mRNAs were upregulated in CD samples and were associated with pediatric CD progression.

A bioinformatics approach was used to intersect DEGs and genes with the highest clinical significance and module eigengene-based connectivity to determine a risk signature. The current novel bioinformatics study combined WGCNA, DEGs and risk signature, taking into account gene interaction network and expression profiles.

We acknowledge some deficiencies in the present study. Publicly available gene profile data were used, and the sample size of the external clinical cohort was small. Only *APCDD*1 was verified in our predictive risk signature, and other predictor mRNAs require validation. *In vivo* and *in vitro* experiments are also required to supplement the IHC analysis, such as Western blotting and qPCR to assess protein expression and transcription rate of the other five predictors in HCT116 and HT29 cell lines.

## 5 Conclusion

A predictive risk signature for the pediatric CD was constructed by bioinformatics methods based on six mRNAs to assist with the stratification and diagnosis of pediatric CD. Further investigation is required to combat pediatric CD.

## Supplementary Material

supplementary material
