# Supplementary material for: Predicting pediatric Crohn's disease based on six mRNA-constructed risk signature using comprehensive bioinformatic approaches
Source: Open Life Sci. 2023 Oct 5;18(1):20220731. doi: 10.1515/biol-2022-0731 (PMC10557890; doi:10.1515/biol-2022-0731)
Supplement: supplementary material [file biol-2022-0731-sm.pdf]

Supplementary material

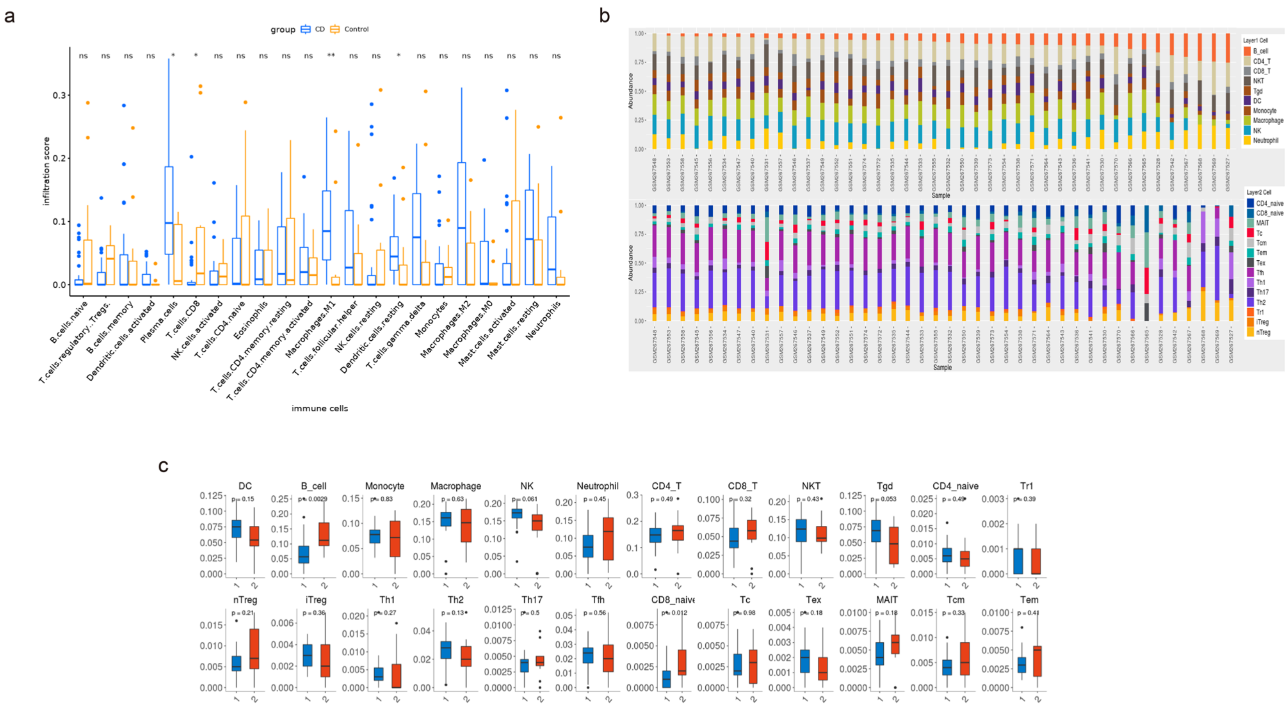

Figure S1: Immune cell infiltration analysis of the dataset GSE10616. (a) The abundance of 22 immune cell types infiltrated in the CD and the control group analyzed by Cibersortx. (b) The proportion of 24 immune cell types in 42 samples computed by ImmuCellAi. (c) The relative abundances of 24 immune cells types in the CD and the control group calculated by ImmuCellAi. In the X axis, 1 represented the CD group, and 2 represented the control group. ns, not significant; *$p < 0.05$; **$p < 0.01$. NKT cells, natural killer T cells; Tgd, γδ T cells; DC, dendritic cells; NK cells, natural killer cells; CD4 naive, naive CD4+ T cells; CD8 naive, naive CD8+ T cell; MAIT, mucosal-associated invariant T cells; Tc, cytotoxic T cells; Tcm, central memory T cells; Tem, effector memory T cells; Tex, exhausted T cells; Tfh, follicular helper T cells; Th1, T helper cells type 1; Th17, T helper cells type 17; Th2, T helper cells type 2; Tr1, type 1 regulatory T cells; iTreg, induced regulatory T cells; nTreg, natural regulatory T cells.

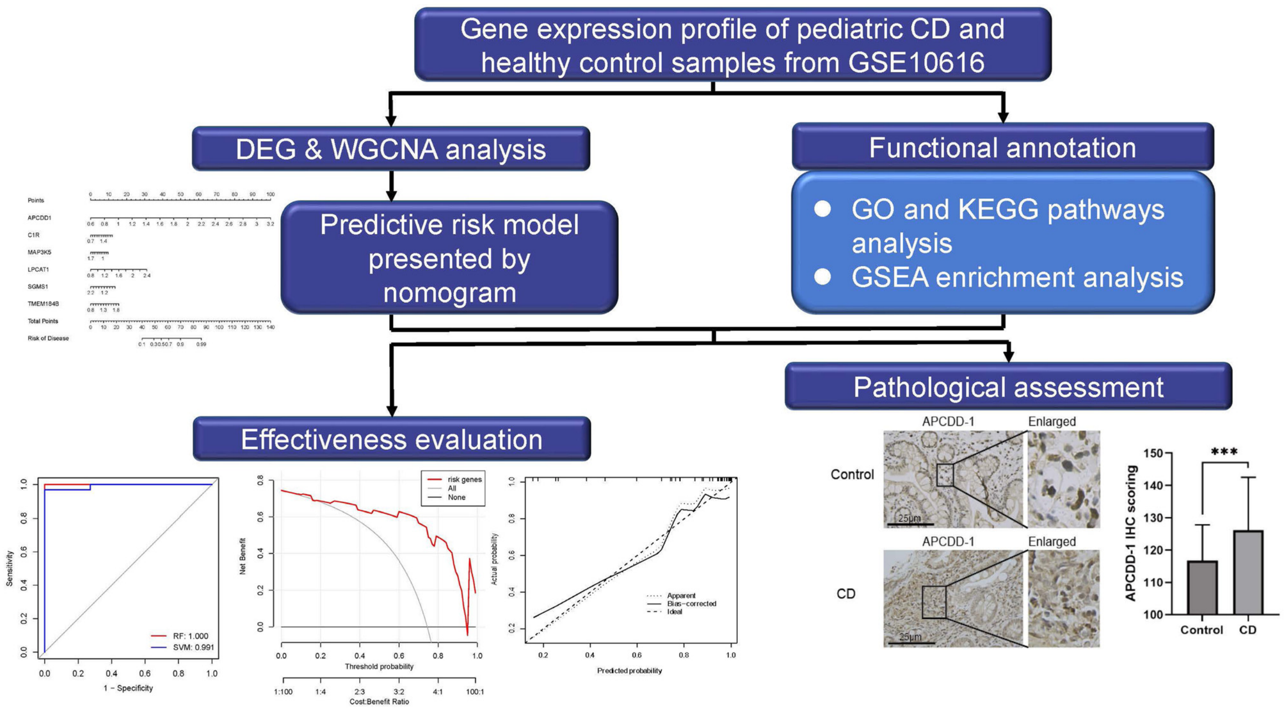

Figure S2: Graphical abstract.

Table S1: Patient sample information

Patient characteristics	Crohn's disease	Control
Sample number	5	5
Gender	Male 3 (60%)	
Female 2 (40%)	Male 2 (40%)	
Female 3 (60%)		
Age	1~18	1–18

Table S2: Characteristic of the GSE10616 microarray datasets

**Total number of patients**	**58**
participants	Colon-only CD 14
Ileo-colonic CD 18	
Ulcerative colitis 10	
Internal control	5
Healthy control 11	
gender	Not determined
platform	GPL5760 (identical to GPL570)
Analysis type	array
Online address	https://www.ncbi.nlm.nih.gov/geo/query/acc.cgi?acc=GSE10616

Table S3: The 283 hub genes in the module midnightblue

ABI2	CHST15	FBN1	LHFP	PEA15	STK10
ACO2	CHST2	FCER1G	LMCD1	PECAM1	STK38
AEBP1	CHSY1	FCHSD2	LOXL1	PHTF1	STON1
AFG3L2	CLEC2B	FJX1	LPCAT1	PIM2	SUCLG1
AGT	CLIP4	FLRT2	LPHN2	PIP4K2A	SUPV3L1
AHR	COL15A1	FNDC1	LRRC8C	PKD2	SYT11
AIMP2	COL1A1	FOXF1	LSAMP	PLEKHO1	TAF5
AKAP1	COL1A2	FSTL1	LSM4	PLSCR3	TCEA3
ALDH1A3	COL3A1	GALNT2	LTBP2	PLXND1	TCEAL7
ANGPTL2	COL4A1	GIMAP2	LUM	PPM1K	TDO2
ANXA1	COL4A2	GIMAP4	LY96	PREX1	TGFBI
ANXA5	COL5A1	GIMAP8	LYZ	PRKAR2B	THY1
APCDD1	COL5A2	GLIPR1	MAB21L2	PRKCH	TIMP1
ARHGEF6	COQ9	GLT8D2	MAN1C1	PRKD1	TIMP2
ARL4C	COX4I1	GMFG	MAP3K5	PRPS1	TLR1
ARNTL2	COX5B	GNA14	MFAP4	PRR16	TMEM154
ASAM	COX6B1	GNA15	MGST3	PTGDR	TMEM158
ASAP1	COX8A	GNS	MMP12	PXDN	TMEM184B
ATP5B	CREB3L2	GOT1	MRPL2	PXMP2	TMEM45A
ATP5I	CS	GPC6	MRPL34	RAB31	TMTC1
ATP5L	CSF1R	GPD1L	MRPS22	RAB8B	TNFRSF1B
BAG2	CSGALNACT2	GPR124	MRPS25	RCBTB1	TPST1
BHLHE40	CSRP2	GPR137B	MSN	RHOJ	TRAM1
BLVRA	CTSK	GPR65	MTCH2	RHOQ	TRIM8
BNC2	CXCR7	GPX7	MXRA5	RILPL2	TRPS1
BOC	CYC1	GRK5	MYO1F	RPS23	TRUB2
BST2	CYSLTR1	GUCY1B3	NCKAP1L	RSPO3	TSHZ3
C12orf23	DAPP1	HHEX	NDUFA1	S1PR1	TUBA1A
C12orf24	DENND5A	HIVEP2	NDUFA10	SACS	TUBB6
C12orf62	DNAJB9	HLA.DRA	NDUFA11	SAMSN1	TXN2
C13orf15	DOCK8	HLA.DRB1	NDUFA2	SDC2	TXNDC15
C1R	DPYSL2	HOMER1	NDUFA6	SDHB	UBAC1
C1orf216	DPYSL3	HSPA13	NDUFA8	SELP	UBASH3B
C3	DUSP14	HTRA1	NDUFAB1	SERPINF1	UQCR11
C3AR1	ECHS1	IFI16	NDUFB10	SESTD1	UQCRFS1
C6orf145	EFEMP2	IFI30	NDUFB8	SGMS1	UQCRQ
C9orf21	EHD3	IGDCC4	NDUFB9	SH2B3	VASN
CALCRL	EIF3K	IGFBP7	NDUFS3	SLC24A3	VAT1L
CARD6	EMP3	IMPA2	NID1	SLC25A4	VEGFC
CCDC51	ETHE1	JAZF1	NXN	SLC2A3	VIM
CCL2	EVI2B	KAL1	OAZ2	SLC40A1	ZCCHC24
CCR1	F2R	KDELC1	OLFML1	SLFN12	ZFP36L1

(Continued)

Table S3: *Continued*

ABI2	CHST15	FBN1	LHFP	PEA15	STK10
CD81	FADS1	KLF5	OLFML2B	SNCAIP	ZNF521
CD93	FAM110B	LAMC1	PAPLN	SNN	
CDH11	FAM114A2	LAPTM5	PAPSS1	SORCS2	
CENPV	FAM20C	LCP1	PCDH18	SPARC	
CH25H	FAM49A	LCP2	PCNX	SRGN	
CHCHD10	FAM89A	LGALS4	PCOLCE	ST8SIA1	

Table S4: The *P*.Value, logFC and the average gene expression rate of *APCDD1*, *C1R*, *MAP3K5*, *LPCAT1*, *SGMS1*, and *TMEM184B*

	p Value	logFC	Average gene expression rate
APCDD1	0.0005760	1.808246719	2.345672008
C1R	0.0002014	1.415161011	2.053143061
MAP3K5	0.0029510	0.603584577	1.449179227
LPCAT1	0.0048330	0.810003029	1.602792887
SGMS1	0.0055229	0.922643451	1.686618375
TMEM184B	0.0008257	0.312445986	1.232517885

Table S5: The top 20 enriched pathways and relevant genes analyzed by GSEA in the pediatric CD and healthy control group in dataset GSE10616

Name	Size	ES	NES	NOM *p*-val	FDR *q*-val
Huntingtons_disease	106	−0.46417	−2.2249	0	9.38×10^{-04}
Cardiac_muscle_contraction	49	−0.52733	−2.16787	0	0.001324
Oxidative_phosphorylation	77	−0.45646	−2.08344	0	0.002908
Parkinsons_disease	74	−0.4519	−2.03613	0	0.003929
Alzheimers_disease	94	−0.41935	−1.9618	0	0.007019
Fatty_acid_metabolism	21	−0.58947	−1.95074	0	0.007165
Citrate_cycle_tca_cycle	19	−0.6111	−1.94676	0	0.006141
Butanoate_metabolism	17	−0.61412	−1.94255	0	0.005835
Proximal_tubule_bicarbonate_reclamation	16	−0.60687	−1.87411	0.003454	0.01046
Propanoate_metabolism	17	−0.56606	−1.77992	0.005338	0.026824
Valine_leucine_and_isoleucine_degradation	22	−0.52307	−1.77008	0.00692	0.026693
Taste_transduction	34	−0.42232	−1.61289	0.014787	0.094595
Aminoacyl_trna_biosynthesis	15	−0.52531	−1.59364	0.022472	0.100298
Homologous_recombination	17	−0.48616	−1.53455	0.031136	0.147713
Aldosterone_regulated_sodium_reabsorption	20	−0.43137	−1.45077	0.063943	0.244149
Olfactory_transduction	58	−0.33996	−1.43175	0.036842	0.259059
Steroid_hormone_biosynthesis	29	−0.38181	−1.3874	0.078571	0.319964
Amyotrophic_lateral_sclerosis_als	26	−0.39019	−1.37692	0.092453	0.321507
Arginine_and_proline_metabolism	32	−0.36535	−1.32661	0.095668	0.407281
Drug_metabolism_cytochrome_p450	37	−0.33268	−1.29884	0.114603	0.452545
2. The top 20 enriched pathways and relevant genes analyzed by GSEA in the healthy control group					
**Name**	**SIZE**	**ES**	**NES**	**NOM p−val**	**FDR q−val**
Cytokine_cytokine_receptor_interaction	159	0.496173	2.629071	0	0
Leishmania_infection	31	0.674989	2.532606	0	0
Nod_like_receptor_signaling_pathway	35	0.605072	2.363963	0	0
Graft_versus_host_disease	17	0.723134	2.356892	0	0
Systemic_lupus_erythematosus	29	0.627143	2.335557	0	0
Chemokine_signaling_pathway	102	0.477864	2.329973	0	0
Cell_adhesion_molecules_cams	64	0.509717	2.288602	0	1.61×10^{-04}
Intestinal_immune_network_for_iga_production	27	0.608103	2.212077	0	2.82×10^{-04}
Hematopoietic_cell_lineage	43	0.545996	2.186916	0	2.51×10^{-04}
Type_I_diabetes_mellitus	19	0.651267	2.161073	0	4.34×10^{-04}
Protein_export	17	0.651469	2.094414	0.002217	9.20×10^{-04}
Asthma	15	0.664655	2.063221	0	0.001407
Allograft_rejection	17	0.636768	2.05288	0	0.001482
Ecm_receptor_interaction	51	0.472849	1.983965	0	0.003035
Viral_myocarditis	30	0.524929	1.976594	0.002169	0.003347
Prion_diseases	21	0.566487	1.971316	0.002299	0.003355
Complement_and_coagulation_cascades	36	0.508486	1.969693	0	0.003222
Glycosaminoglycan_biosynthesis_chondroitin_sulfate	17	0.616311	1.968034	0	0.003043

(Continued)

Table S5: *Continued*

Name	Size	ES	NES	NOM *p*-val	FDR *q*-val
Toll_like_receptor_signaling_pathway	60	0.440674	1.957068	0	0.003341
Antigen_processing_and_presentation	34	0.499			
178	1.918852	0.002119	0.004919		
